# Capitation-Based Financing Hampers the Provision of Preventive Services in Primary Health Care

**DOI:** 10.3389/fpubh.2016.00200

**Published:** 2016-09-13

**Authors:** János Sándor, Karolina Kósa, Magor Papp, Gergő Fürjes, László Kőrösi, Mihajlo Jakovljevic, Róza Ádány

**Affiliations:** ^1^Department of Preventive Medicine, Faculty of Public Health, University of Debrecen, Debrecen, Hungary; ^2^Faculty of Public Health, Institute of Behavioural Sciences, University of Debrecen, Debrecen, Hungary; ^3^National Institute of Health Development, Budapest, Hungary; ^4^National Health Insurance Fund, Budapest, Hungary; ^5^Department of Health Economics and Pharmacoeconomics, Faculty of Medical Sciences, University of Kragujevac, Kragujevac, Serbia; ^6^MTA-DE Public Health Research Group, Department of Preventive Medicine, Faculty of Public Health, University of Debrecen, Debrecen, Hungary

**Keywords:** capitation-based financing, primary health care, preventive services, reorientation of primary care, health status assessment

## Abstract

Mortality caused by non-communicable diseases has been extremely high in Hungary, which can largely be attributed to not performed preventive examinations (PEs) at the level of primary health care (PHC). Both structures and financial incentives are lacking, which could support the provision of legally defined PEs. A Model Programme was launched in Hungary in 2012 to adapt the recommendations for PHC of the World Health Organization. A baseline survey was carried out to describe the occurrence of not performed PEs. A sample of 4320 adults representative for Hungary by age and gender was surveyed. Twelve PEs to be performed in PHC as specified by a governmental decree were investigated and quantified. Not performed PEs per person per year with 95% confidence intervals were computed for age, gender, and education strata. The number of not performed PEs for the entire adult population of Hungary was estimated and converted into expenses according to the official reimbursement costs of the National Health Insurance Fund. The rate of service use varied between 16.7 and 70.2%. There was no correlation between the unit price of examinations and service use (*r* = 0.356; *p* = 0.267). The rate of not performed PEs was not related to gender, but older age and lower education proved to be risk factors. The total number of not performed PEs was over 17 million in the country. Of the 31 million euros saved by not paying for PEs, the largest share was not spent on those in the lowest educational category. New preventive services offered in the reoriented PHC model program include systematic and scheduled health examination health promotion programs at community settings, risk assessment followed by individual or group care, and/or referral and chronic care. The Model Programme has created a pressure for collaborative work, consultation, and engagement at each level, from the GPs and health mediators up to the decision-making level. It channeled the population into preventive health services shown by the fact that more than 80% of the population in the intervention area has already participated in the health status assessment.

## Introduction

The health status of the population of Hungary has been much less favorable compared to the EU15 countries, that is, those which joined the European Union before May 2004. Even though life expectancy at birth increased by about 5 years, premature mortality due to diseases of the circulatory system decreased by 35% between 1980 and 2008 (1), due to malignant neoplasms decreased by 14% between 2004 and 2013, the gap between Hungary and the EU15 increased in this decade (2). Life expectancy in Hungary at age 65 was 3.39 years less in 2004 that increased to 3.59 years less in 2013 compared to the EU15 average (3).

Considering the firmly established relationship between preventive health services and mortality (4), it is safe to state that the comparatively high premature mortality in Hungary can be partly attributed to insufficient preventive services in primary health care (PHC). According to the last health survey in Hungary, 17% of young adults, 26% of those of middle age, and 23% of the elderly did not have access to health services, even though they would have needed them (5). Not provided preventive services are unaccounted for by the lack of health policy since a ministerial decree has been in effect for two decades detailing all preventive examinations (PEs) and checkups for specific age groups at specified intervals that should be performed by general practitioners (GPs). These services are free for patients and paid for by health insurance, so one potential explanation for not providing them might be their high cost to the provider and/or the insurer (6).

Hungary maintains a single payer insurance-based health-care system with practically universal coverage that is managed by the state-owned National Health Insurance Fund. The most important problems of the health system include underperformance – a rather poor population health status that does not match the economic performance of the country (7, 8); so-called “gratitude payments,” informal out-of-pocket payments to service providers (9) that developed decades ago (10); understaffing coupled with an internal and international migration of physicians (11, 12); as well as allocative inefficiency coupled with/due to unstable and non-transparent stewardship (13–15). Dysfunctions of the system have been recognized and analyzed by a number of respected national and international authors, and virtually every government in the past two decades attempted to introduce smaller or larger scale reforms to remedy the situation with limited results (16–18), in part due to underfunding that has been below average compared to OECD countries (19). Capitation-based reimbursement system was introduced for financing general practices of primary care in 1992, based on the number of registered (insured) inhabitants (as a multiplier). Due to inherent nature capitation-based financing, the level of reimbursement does not depend on the quantity and/or quality of the provided services. Rather, the GP is financially motivated to maximize the number of persons in the practice and minimize performance.

A rare opportunity to reorient the health-care system emerged in 2006, some years after the EU accession, when Switzerland committed 1 billion CHF to help reduce the economic and social disparities in the 10 new EU member states (20). Of this amount, 131 million CHF (the third largest sum) was allocated to Hungary in the framework of the Swiss-Hungarian Cooperation Programme designating 5 thematic and 2 geographical focus areas (21). Subsequently, both the Swiss and Hungarian Governments designated agencies for the implementation of the Cooperation in 9 specific priority areas between 2008 and 2012 and approved altogether 39 project plans for implementation (22). In the priority area of developing human resources and society, a community-oriented model program for PHC and disease prevention has been implemented between July 2012 and June 2016 in four geographical areas.

A consortium of nine institutional members led by the National Centre for Health Care Provision was set up, including all four universities of Hungary that provide medical education: the Universities of Debrecen, Pécs, Szeged, and Semmelweis University of Budapest; the Hungarian Scientific Association of General Practitioners, the National Institute of Primary Health Care, the National Health Insurance Fund, and the Hungarian Association of Health Visitors. Nine work packages (WPs), including experts from members of the consortium, were set up to work on various aspects of the program. A Board of Supervision was created in which experts in leading positions from the consortium member institutions and representatives of the Swiss Government were delegated.

The program titled “Public Health Focused Model Programme for Organising Primary Care Services Backed by a Virtual Care Service Centre” (23) conceptually aimed at testing a new model of primary care and providing evidence to the large-scale restructuring of the health-care system in Hungary. The model extends the renewal of the PHC system both in terms of its human resources and its services (24). The Model Programme was based on the principles recommended by WHO – except universal coverage which has already been achieved (25).

**Figure 1:**
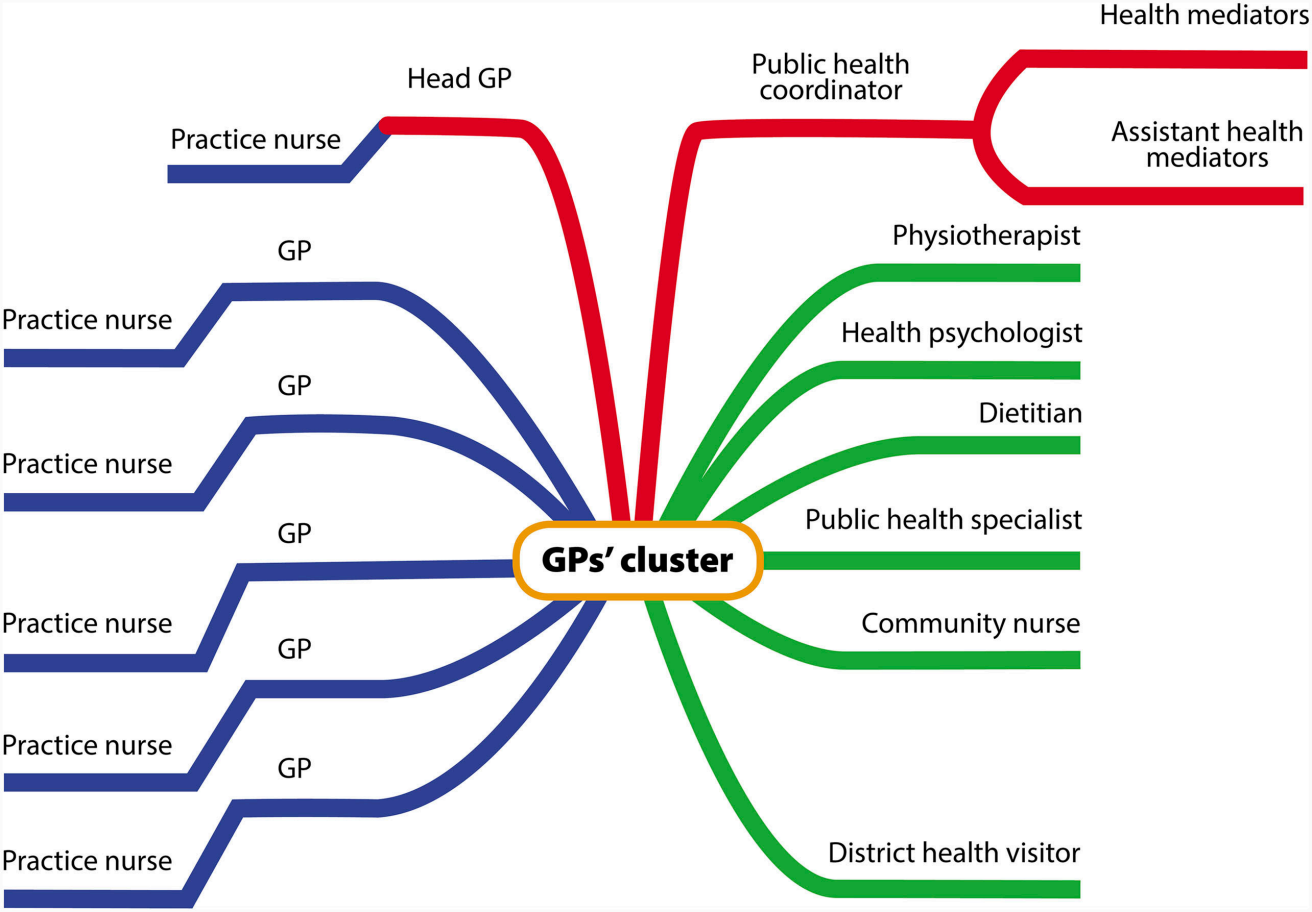
**Human resources of one GPs’ cluster**.

General practitioners working alone and helped only by practice nurses were invited to form “GPs’ clusters” in 2012 along with other health professionals. The composition of the cluster (shown in Figure 1) was described elsewhere (26).

Briefly, six GPs, each employing one practice nurse, formed one GP cluster that employs other health professionals with BSc or MSc degrees, such as dietitian, physiotherapist, health psychologist, community nurse (1 of each in each cluster), public health specialists (2 in each cluster), and health workers with vocational or *ad hoc* training (health mediators – 12 in each cluster). Health visitors (independent health workers charged with looking after children, expecting mothers and mothers with children), though not employed by the clusters, also collaborate with cluster workers. Altogether, 4 GP clusters including 24 GPs were created in 4 areas of Hungary, each of which is located in the 2 most disadvantaged regions of the country. The Model Programme started to provide new services by the Fall of 2014 after preparing the protocols, staffs, and infrastructures.

The study investigated the frequency of and risk factors for not-performed PHC level preventive services. The hypothesis of the present study is that not performed preventive services are more frequent in younger age groups, males, and those having lower education, and more frequent in regular primary care. Another hypothesis states that preventive services are not performed due to their high cost to the provider and/or insurer.

## Materials and Methods

### Preventive Services Investigated

Preventive services were defined as those contributing to the prevention of leading causes of non-communicable disease morbidity and mortality as specified in the 51/1997 (XII.18.) Decree of the Ministry of Welfare for adults (6). All the 12 services/examinations and their prescribed frequency are presented in Table 1. These services are performed by GPs at the request of the insured person.

**Table 1:** **Examinations for the prevention of various non-communicable diseases and their unit price according to cross-sectional data collection in 2012**.

A	B	C	D	E	F	G

	Preventive examination	Unit price in euro^a^	Frequency of examination as legally specified^b^	Percent of examinations performed as legally specified^b^	Percent of examinations not performed as legally specified^b^	Percent of examination not completed in 1 year in relation to the specified time period
1	General health check	3.27	Once every 3 years	37.8% [36.3–39.3]	62.2% (60.7–63.7)	20.7% (20.2–21.2)
2	Fasting blood glucose measurement	0.27	Once every 2 years	66.1% [64.7–67.6]	33.9% (32.4–35.3)	17.0% (16.2–17.7)
3	Urine test	2.13	Once every 2 years	49.1% [47.5–50.6]	50.9% (49.4–52.5)	25.5% (24.7–26.3)
4	Serum creatinine measurement	0.29	Once every 2 years	38.3% [36.8–39.8]	61.7% (60.2–63.2)	30.9% (30.1–31.6)
5	Serum lipid profile	1.64	Once every 2 years	57.6% [56.0–59.1]	42.4% (40.9–44.0)	21.2% (20.5–22.0)
6	Screening for hearing loss	0.26	Once every year over 65 years of age	16.7% [14.2–19.2]	83.3% (80.8–85.8)	83.3% (80.8–85.8)
7	Screening for visual acuity	0.40	Once every year over 65 years of age	36.9% [33.7–40.1]	63.1% (59.9–66.3)	63.1% (59.9–66.3)
8	Nutritional assessment and counseling	1.87	Once every 3 years	26.5% [25.2–27.9]	73.5% (72.1–74.8)	24.5% (24.0–24.9)
9	Peripheral atherosclerosis checkup	2.62	Once every 3 years over 40 years of age	26.4% [24.7–28.0]	73.6% (72.0–75.3)	24.5% (24.0–25.1)
10	Screening for oral cancer	1.94	Once every 3 years	21.7% [20.5–23.0]	78.3% (77.0–79.5)	26.1% (25.7–26.5)
11	Cervical cancer screening^c^	5.13	Once every 3 years between 25 and 65 years of age in females	70.2% [67.9–72.6]	29.8% (27.4–32.1)	9.9% (9.1–10.7)
12	Breast cancer screening^c^	13.79	Once every 2 years between 45 and 65 years of age in females	56.1% [52.6–59.6]	43.9% (40.4–47.4)	22.0% (20.2–23.7)

*^a^Reimbursement to service providers by the National Health Insurance Fund*.

*^b^The 51/1997. Decree of the Ministry of Welfare specifies the regularity of each preventive examination listed in the table for age groups*.

*^c^GP expected to motivate and register the participation in screening performed by outpatient institution*.

### Sources and Collection of Data

A baseline survey was carried out to describe both the health status of the general population and that of the population in the area of the Model Programme before new services were offered; i.e., data collection was carried out in two populations. One of them – representative for the general population – was based on a nationwide network of GPs (*N* = 131) participating in the General Practitioners’ Morbidity Sentinel Stations Programme (GPMSSP), a large-scale health monitoring network established in 1998 (27). Another population was comprised of those to be serviced by the Model Programme. Data collection in this population was carried out involving all GPs who participate in the Model Programme (*N* = 34). Client samples from both populations were combined for the present analysis. A list of all adults was provided by each GP. Pooling of all lists provided the sampling frame of which 4320 adults were selected randomly. The pool of participants was ultimately restricted to those aged at least 20 years old in order to make it comparable to the data of Census 2011 during data analysis.

The use of preventive services in PHC was surveyed using a questionnaire. Nurses employed in the practices were trained for administering the questionnaire and served as interviewers. Data collection had been carried out between December 2012 and July 2013.

The unit prices in euros of preventive services reimbursed to service providers in the year 2012 were given by the National Health Insurance Fund. Census 2011 population data were acquired from the Hungarian Central Statistical Office.

### Questionnaire

The questionnaire had items to determine age, sex, and educational level (primary school or less, vocational school, high school, or tertiary education) as a proxy for socio-economic status. The use of preventive services was investigated by sex, educational level, and age groups (20–39; 40–64; and over 65 years). Among participants, distinction was made between those who were screened as prescribed in the governmental decree (that is, had been assessed for family history, oral cancer screening, atherosclerosis checkup, nutritional habits within 3 years, or for serum lipid, serum glucose, serum creatinine measurement and urine check within 2 years, and screening for hearing loss and visual acuity yearly among 65 or older, those between 25 and 64 participated in cervical cancer screening in 3 years, those between 45 and 64 participated in breast cancer screening in 2 years), and those who were not. Responders were classified as regular service users if they attended services at the recommended frequency or service users at a lower-than-recommended frequency if not.

### Data Analysis

Preventive service delivery rate was defined as the proportion of adults who used services at the recommended frequency and was calculated separately for each service, specified by age groups, sex, and level of education. Proportions were compared by their 95% confidence intervals.

The non-completion of preventive services was calculated taking into account the varying frequency of PEs. The proportion of adults who had received the service was based on the data of the questionnaire survey. Based on the proportion of performed services, calculations were made to establish the proportion of those who did not receive the service. The proportion of clients who had not received a particular examination was divided by the number of years within which the examination should be performed, yielding the percentage of population who did not receive the specific examination per year.

Using the Census 2011 population data, the number of adults who did not receive a specified preventive service was estimated for the whole country. Considering the recommended frequency of service use, the number of not implemented preventive service delivery was computed for 1 year for each service for the Hungarian population aged at least 20 years, and the number of not delivered services per person per year had also been determined. The unit price of preventive services from the reimbursement list of National Health Insurance Fund was applied to determine the avoided cost of not delivered services per year per person in the Hungarian population.

In order to test our hypothesis that PEs are not performed due to their high cost to the provider and/or insurer, correlation between the unit price of each service and its delivery was investigated for all 12 services in the total sample and in various educational categories.

This study was carried out with the permission of the Ethical Committee of the Hungarian National Scientific Council on Health (number of permission: TUKEB 57097/2012/EKU and TUKEB 2213-6/2013/EKU). All subjects gave written informed consent in accordance with the Declaration of Helsinki.

## Results

The unit prices (column C in Table 1) of the investigated preventive services (column B in Table 1) varied in a wide range: they were less than 1 euro for 4 examinations, more than 1 and less than 4 euros for 6 examinations, and more than 5 but less than 14 euros for 2 examinations (cervical and breast cancer screening).

The proportion of clients in column G in Table 1 shows the percentage of population who did not receive the specific examination per year. The highest proportion of clients did not receive hearing loss examination (83.3%) and visual acuity checkup (63.1%). The lowest proportions of services not provided were cervical cancer screening (9.9%) and fasting blood glucose measurement (17.0%).

**Figure 2:**
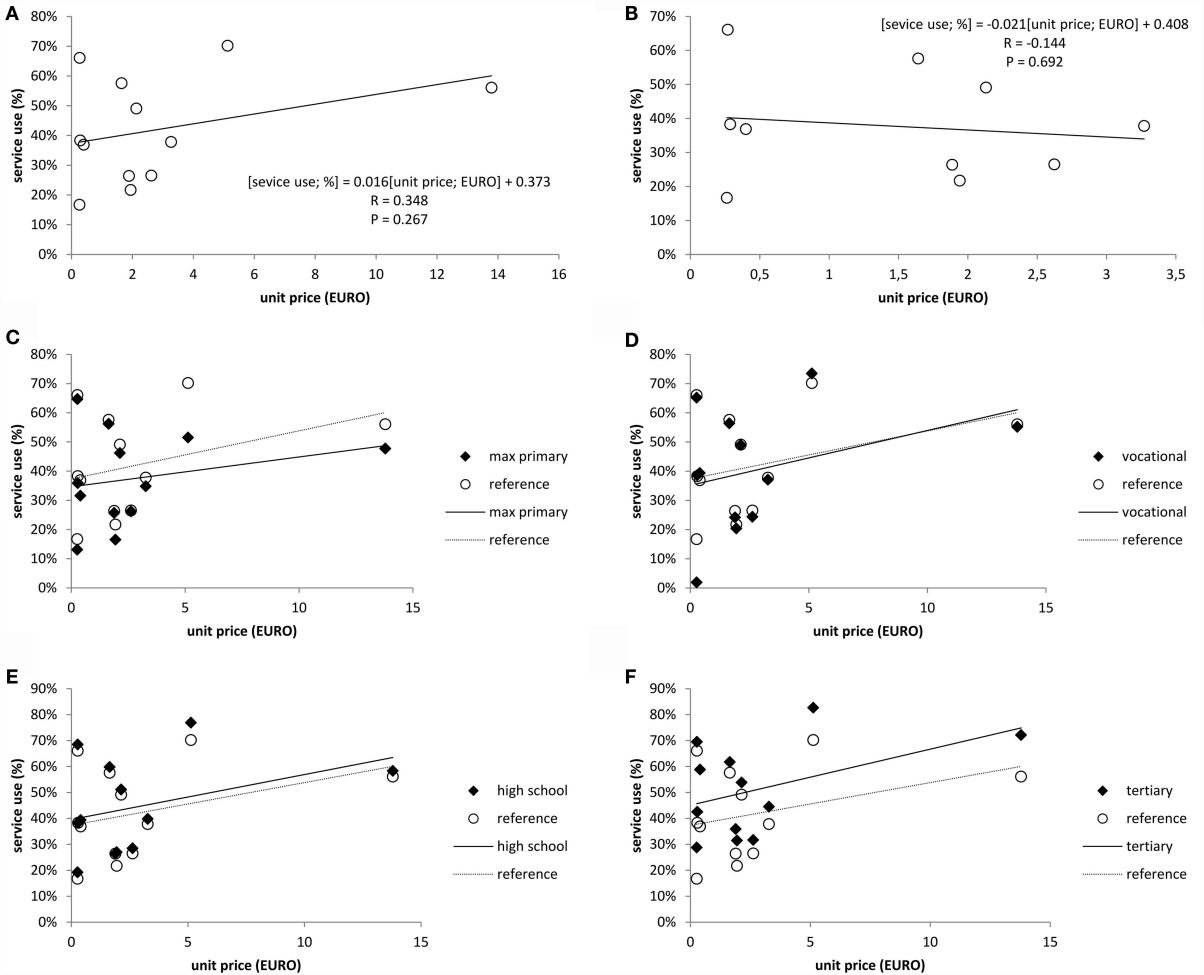
**Correlation between the unit price of examination and frequency of all performed examinations (A), performed examinations trimmed for cervical and breast cancer screenings (B), and all performed examinations by education (C–F)**. Footnote: reference data and trends represent the service uses in the studied sample in respective of education level.

Correlation between the unit price of services (column C in Table 1) and service use (column E in Table 1) for all 12 services (Figure 2A) showed a non-significant correlation (*r* = 0.356, *p* = 0.267). To eliminate the distorting effect of the two highest priced examinations (cervical and breast cancer screening), the correlation was repeated after removing data points for these two examinations. This did not change the conclusion that unit price of PEs is not related to service use (*r* = −0.144, *p* = 0.692) as shown in Figure 2B.

Non-performed examinations were also analyzed by the educational accomplishments of clients. Results are shown in Figures 2C–F revealing that in comparison to the reference group comprised by all adults, those with tertiary education used preventive services much more frequently, while those with no higher than primary education were less likely to use.

Further dissecting our data, not performed PEs were stratified by age group, gender, and educational levels as shown in Table 2. The rates of not performed examinations were higher among women in each educational category and almost each age group, except the youngest one (20–39 years). However, these gender differentials are not significant as reflected by the overlapping confidence intervals. The rates of not performed examinations were highest among those with the lowest education, whereas they were the least among those with higher educational accomplishments. The educational differential between the lowest and highest educational category in terms of not performed preventive services proved to be significant. The rate of not performed PEs increases by age, and it is highest in those over 65 years of age in each educational category, probably reflecting – among others – a large share of unserviced needs in the oldest age group. Not performed preventive services were analyzed by dividing the total sample to those who represented the general population (data provided by the GPMSSP) and to those who represented the population of the Model Programme. Further stratifying these two samples by age group and education, the intriguing pattern of the youngest age group having the lowest rate of non-performed services, regardless of education, was detected here again (Table 3). The population of the Model Programme in most subgroups has higher unserviced needs, regardless of age and educational categories, compared to the general population. There is one notable exception from this, namely, the youngest age groups (20–39 years) with high school and higher education diplomas. Of all age groups, those above the age of 65-year age group of the population of the Model Programme have the highest unserviced needs, which is 1.7–2 times higher than that of the younger age groups in all educational categories. In contrast, the oldest age group of the general population has unserviced needs only 1.3–1.6 times higher across the educational categories.

**Table 2:** **The number of not performed preventive examinations per person per year (with the corresponding 95% confidence interval) and yearly number of not performed preventive examinations (estimated for the whole population of Hungary) by age group, gender, and education**.

	Completed primary education or less		Vocational training		High school		Higher education	
	Male	Female	Male	Female	Male	Female	Male	Female
20–39 years	2.15 [1.93–2.37] (489416)	1.98 [1.72–2.25] (361539)	2.05 [1.91–2.19] (877005)	1.78 [1.58–1.97] (435450)	1.96 [1.7–2.22] (989936)	1.76 [1.52–2.01] (959006)	1.91 [1.61–2.21] (505557)	1.76 [1.52–2.01] (2439450)
40–64 years	2.11 [1.89–2.33] (590225)	2.33 [2.09–2.56] (1080200)	1.89 [1.76–2.02] (1272900)	2.08 [1.88–2.28] (747913)	1.91 [1.59–2.23] (780705)	1.93 [1.67–2.2] (1198935)	1.76 [1.44–2.09] (468488)	2.03 [1.78–2.29] (3623447)
65 years and above	3.2 [2.78–3.62] (1035063)	3.38 [3.14–3.62] (2491206)	2.88 [2.44–3.32] (158396)	3.21 [2.75–3.67] (93193)	3.29 [2.36–4.22] (409246)	2.77 [2.24–3.3] (560110)	2.34 [1.63–3.06] (269468)	3.18 [2.83–3.53] (3370039)

Total (gender)	2.55 [2.25–2.84] (2114704)	2.84 [2.6–3.08] (3932946)	2.0 [1.84–2.15] (2308301)	2.02 [1.8–2.23] (1276556)	2.1 [1.74–2.46] (2179887)	1.99 [1.69–2.29] (2718052)	1.93 [1.54–2.31] (1243513)	2.23 [1.96–2.51] (9432936)

Total (all)	2.73 [2.47–2.99] (6047650)		2.0 [1.83–2.18] (3584858)		2.04 [1.71–2.36] (4897939)		1.85 [1.48–2.21] (2748896)	

**Table 3:** **The number of not performed preventive examinations per person per year (with the corresponding 95% confidence interval) and yearly number of not performed preventive examinations by age group and education in the general population and in the population of the intervention area of the Model Programme**.

	Completed primary education or less		Vocational training		High school		Higher education	
	General population	Population of the Model Programme	General population	Population of the Model Programme	General population	Population of the Model Programme	General population	Population of the Model Programme
20–39 years	2.08 [1.84–2.32] (850955)	2.06 [1.62–2.44] (228)	1.95 [1.79–2.11] (1312455)	1.96 [1.6–2.29] (298)	1.86 [1.61–2.11] (1948942)	1.72 [1.17–2.25] (114)	1.75 [1.47–2.03] (1189012)	1.65 [0.92–2.34] (61)
40–64 years	2.25 [2.02–2.47] (1670425)	2.43 [1.96–2.85] (295)	1.96 [1.8–2.11] (2020813)	2.12 [1.76–2.45] (425)	1.92 [1.64–2.21] (1979640)	2.05 [1.35–2.7] (107)	1.77 [1.44–2.09] (1064886)	1.93 [1.17–2.66] (85)
65 years and above	3.32 [3.03–3.62] (3526269)	3.69 [3.1–4.2] (462)	2.99 [2.54–3.44] (251589)	3.55 [2.53–4.34] (185)	2.97 [2.28–3.65] (969357)	3.31 [1.36–4.51] (46)	2.41 [1.67–3.15] (494998)	3.38 [1.03–4.95] (34)

Total (by area)	2.73 [2.47–2.99] (6047650)	2.76 [2.25–3.19] (985)	2 [1.83–2.18] (3584858)	2.24 [1.8–2.63] (907)	2.04 [1.71–2.36] (4897939)	2.02 [1.26–2.67] (267)	1.85 [1.48–2.21] (2748896)	1.98 [1.06–2.78] (180)

Comparing the groups with the lowest educational accomplishment (completed primary education or less) to those with the highest (higher education), the former group has the highest rate of not performed screening for hearing loss and visual acuity, screening for cervical and breast cancer as well as peripheral atherosclerosis checkup. Those with vocational training also had higher rates of not completed examinations for screening for hearing loss, visual acuity, breast cancer, and peripheral atherosclerosis checkup compared to those with higher education (Table 4).

**Table 4:** **The number of not performed preventive examinations per person per year (with the corresponding 95% confidence interval) and yearly number of not performed preventive examinations (estimated for the whole population of Hungary) by education and preventive examinations**.

	Type of preventive examination	Completed primary education or less	Vocational training	High school	Higher education	Total
1	General health check	0.22 [0.2–0.24] (482889)	0.21 [0.19–0.23] (376458)	0.2 [0.17–0.23] (487523)	0.19 [0.15–0.22] (276891)	0.21 [0.18–0.23] (1623761)
2	Fasting blood glucose	0.17 [0.14–0.2] (370871)	0.18 [0.16–0.21] (327986)	0.16 [0.12–0.21] (396424)	0.16 [0.11–0.2] (232430)	0.17 [0.13–0.2] (1327710)
3	Urine test	0.26 [0.23–0.3] (582351)	0.26 [0.23–0.29] (465513)	0.25 [0.21–0.3] (607373)	0.24 [0.19–0.29] (351439)	0.25 [0.22–0.29] (2006676)
4	Serum creatinine	0.32 [0.28–0.35] (698326)	0.31 [0.29–0.34] (556325)	0.32 [0.27–0.36] (759417)	0.29 [0.24–0.34] (434490)	0.31 [0.27–0.35] (2448557)
5	Serum lipid profile	0.21 [0.18–0.24] (464312)	0.23 [0.2–0.25] (407930)	0.21 [0.16–0.25] (499780)	0.20 [0.15–0.24] (292497)	0.21 [0.17–0.25] (1664519)
6	Screening for hearing loss	0.42 [0.39–0.44] (919483)	0.04 [0.03–0.04] (67683)	0.11 [0.1–0.13] (265436)	0.1 [0.08–0.12] (146394)	0.18 [0.16–0.19] (1398997)
7	Screening for visual acuity	0.33 [0.3–0.36] (725694)	0.03 [0.02–0.03] (49496)	0.08 [0.07–0.1] (204044)	0.06 [0.04–0.08] (85102)	0.13 [0.12–0.15] (1064335)
8	Nutritional assessment and counseling	0.24 [0.23–0.26] (542384)	0.25 [0.24–0.27] (455217)	0.24 [0.21–0.27] (578875)	0.23 [0.2–0.26] (340545)	0.24 [0.22–0.27] (1917022)
9	Peripheral atherosclerosis checkup	0.2 [0.18–0.21] (439546)	0.16 [0.15–0.17] (291056)	0.14 [0.12–0.15] (333321)	0.12 [0.1–0.14] (173261)	0.16 [0.14–0.17] (1237184)
10	Screening for oral cancer	0.28 [0.26–0.3] (617741)	0.27 [0.25–0.28] (475245)	0.25 [0.22–0.27] (589469)	0.23 [0.2–0.26] (341765)	0.26 [0.23–0.28] (2024221)
11	Cervical cancer screening	0.16 [0.14–0.19] (98851)	0.09 [0.07–0.1] (49997)	0.07 [0.05–0.1] (74036)	0.05 [0.03–0.08] (38450)	0.09 [0.07–0.11] (261335)
12	Breast cancer screening	0.26 [0.23–0.29] (105202)	0.22 [0.19–0.25] (61951)	0.21 [0.17–0.25] (102242)	0.14 [0.1–0.18] (35631)	0.21 [0.18–0.25] (305026)

Not performed examinations as shown in Table 4 were calculated in terms of financial expenses that are shown by type of examination and educational level in Table 5. The differences between educational categories in annual expenses per person of not performed examinations were below 1 euro in all examinations apart from breast cancer (even in this case, the difference was less than 2 euro).

**Table 5:** **The expenses in euro of not performed preventive examinations per person per year [with the corresponding 95% confidence interval] and yearly expenses of not performed preventive examinations (estimated for the whole population of Hungary, in million euro/year) by education and preventive examinations**.

	Type of preventive examination	Completed primary education or less	Vocational training	High school	Higher education	Total
1	General health check	0.713 [0.643–0.783] (1.58)	0.688 [0.633–0.743] (1.23)	0.663 [0.567–0.76] (1.59)	0.609 [0.499–0.719] (0.91)	0.673 [0.59–0.755] (5.31)
2	Fasting blood glucose	0.045 [0.037–0.053] (0.1)	0.049 [0.043–0.056] (0.09)	0.044 [0.033–0.056] (0.11)	0.042 [0.03–0.054] (0.06)	0.045 [0.036–0.055] (0.36)
3	Urine test	0.56 [0.49–0.631] (1.24)	0.554 [0.5–0.609] (0.99)	0.538 [0.443–0.634] (1.29)	0.503 [0.399–0.608] (0.75)	0.542 [0.461–0.623] (4.28)
4	Serum creatinine	0.09 [0.081–0.1] (0.2)	0.089 [0.082–0.096] (0.16)	0.091 [0.078–0.103] (0.22)	0.084 [0.07–0.098] (0.12)	0.089 [0.078–0.1] (0.7)
5	Serum lipid profile	0.344 [0.291–0.397] (0.76)	0.374 [0.333–0.416] (0.67)	0.341 [0.27–0.413] (0.82)	0.323 [0.245–0.4] (0.48)	0.346 [0.285–0.407] (2.73)
6	Screening for hearing loss	0.109 [0.104–0.115] (0.24)	0.01 [0.009–0.011] (0.02)	0.029 [0.025–0.033] (0.07)	0.026 [0.021–0.031] (0.04)	0.047 [0.043–0.05] (0.37)
7	Screening for visual acuity	0.131 [0.12–0.142] (0.29)	0.011 [0.009–0.013] (0.02)	0.034 [0.026–0.041] (0.08)	0.023 [0.014–0.031] (0.03)	0.054 [0.046–0.061] (0.43)
8	Nutritional assessment and counseling	0.642 [0.591–0.694] (1.42)	0.667 [0.628–0.706] (1.19)	0.632 [0.56–0.703] (1.52)	0.6 [0.517–0.683] (0.89)	0.637 [0.576–0.697] (5.03)
9	Peripheral atherosclerosis checkup	0.375 [0.346–0.403] (0.83)	0.307 [0.291–0.323] (0.55)	0.262 [0.231–0.292] (0.63)	0.22 [0.185–0.255] (0.33)	0.296 [0.268–0.323] (2.33)
10	Screening for oral cancer	0.542 [0.509–0.574] (1.2)	0.516 [0.488–0.543] (0.92)	0.476 [0.424–0.528] (1.14)	0.446 [0.386–0.506] (0.66)	0.498 [0.455–0.54] (3.93)
11	Cervical cancer screening	0.839 [0.722–0.956] (0.51)	0.449 [0.361–0.537] (0.26)	0.383 [0.276–0.491] (0.38)	0.281 [0.171–0.392] (0.2)	0.468 [0.361–0.574] (1.34)
12	Breast cancer screening	3.602 [3.149–4.055] (1.45)	3.089 [2.68–3.498] (0.85)	2.876 [2.354–3.398] (1.41)	1.925 [1.35–2.501] (0.49)	2.952 [2.462–3.442] (4.21)

As it is shown in Table 6, the total number of not performed examinations was over 17 million based on data from 2012 in a population of 7.8 million adults over the age of 20 years in the country. Of the 31 million euros saved by not paying for PEs, its largest share was not spent on those having the lowest education. Not performed examinations are 25% higher among those with the lowest education. Expenses saved per person per year were 30% higher among those with completed primary education or less.

**Table 6:** **Features of the correlation between the frequency of not performed examinations and unit price of examination in the educational groups (*estimated measures for Hungary in 2012*)**.

	Completed primary education or less	Vocational training	High school	Higher education	Total
Number of not performed examinations (million/year)	6.05 [5.47–6.63]	3.58 [3.28–3.89]	4.90 [4.12–5.68]	2.75 [2.21–3.29]	17.28 [15.07–19.49]
Expenses of not performed examinations (million euro/year)	9.82 [8.82–10.83]	6.96 [6.34–7.57]	9.27 [7.81–10.72]	4.97 [3.98–5.95]	31.01 [26.96–35.06]
Population number by census	2,214,329	1,790,000	2,404,084	1,488,022	7,896,435
Number of not performed examinations/person-year	2.73 [2.47–2.99]	2.00 [1.83–2.18]	2.04 [1.71–2.36]	1.85 [1.48–2.21]	2.19 [1.91–2.47]
Expenses of not performed examinations (euro/person-year)	4.44 [3.98–4.89]	3.89 [3.54–4.23]	3.85 [3.25–4.46]	3.34 [2.68–4.00]	3.93 [3.41–4.44]

### Preventive Services in the Primary Care Model Programme of Hungary

As it is reflected by the data presented above, there has been an enormous need for preventive services in all segments of the Hungarian adult population, fully justifying the reorientation of PHC with a focus on public health. This reorientation was made possible by the extension of primary care teams with non-medical health professionals in the Primary Health Care Model Programme, creating the basis on which preventive services could be extended and provided to all clients (28).

**Figure 3:**
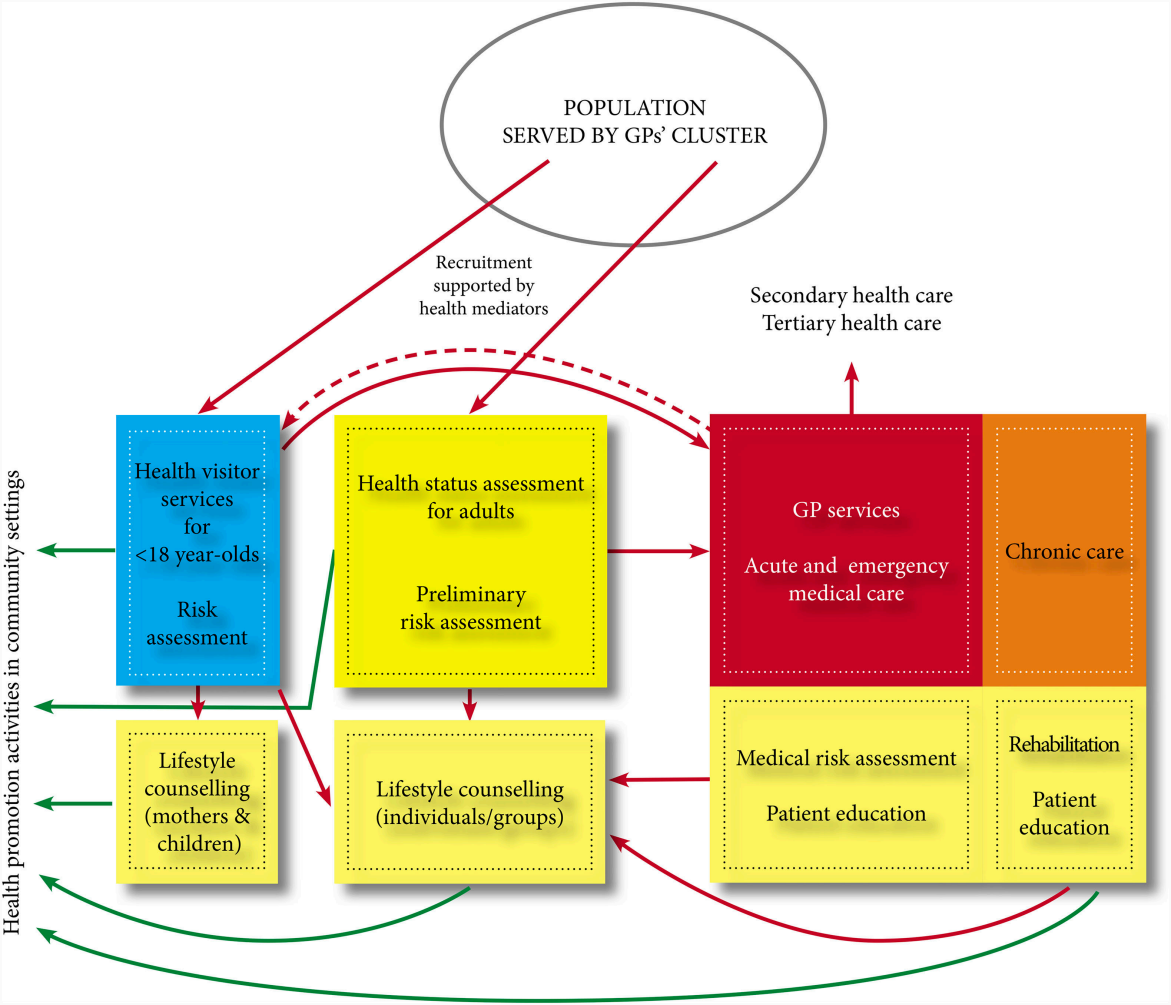
**Services provided by GPs’ cluster**.

Extended services include systematic and scheduled health examination for all clients of GPs regardless of care history, risk assessment followed by individual or group care – including physiotherapy, nutritional and/or psychological interventions, and health education – and/or referral and chronic care if needed based on the result of health examination (see Figure 3). In addition, community-based health promoting programs are offered on an *ad hoc* or regular basis in each participating settlement that were selected so as to involve cities and villages with high proportions of disadvantaged, mostly Roma population groups in economically disadvantaged areas. The single most important outcome of the program will be its conceptual and practical contribution to the reforming of the health-care system as recommended by the World Health Organization by extending this PHC model nationwide.

The five new services systematically provided for all clients in the Model Programme are described below. These services are not available in other regular practices except the health status assessment followed by medical risk assessment. However, assessment must be requested by the client/insured person.

1. *Health promoting activities* in communities of the GPs’ clusters are organized according to a pre-approved plan by the public health specialists. Their aim is to improve the determinants of health and equity in community settings, mostly in schools and workplaces. The targeting of those in greatest need has been facilitated by the abovementioned health mediators; most of them were Roma women recruited from the local communities. Many of these community activities have been organized in collaboration with local stakeholders, including the local governments. Special mother–baby clubs were organized by health visitors and have been offered monthly to low-income mothers with young children.
2. *Health status assessments* are offered to all adults over 18 years of age who belong to the individual practices of the cluster and performed by the public health specialist and the community nurse. The first assessment is used as a baseline against which changes in health status are compared during the program. (Items 1–9 in Table 1 were all parts of the health status assessment.) Depending on the results of the assessment, clients are referred to various preventive services or health promoting programs, or if it is necessary, referred to medical risk assessment carried out by the GPs.
3. *Medical risk assessment* is carried out by GPs to determine the significance of the risk factors and/or conditions, which are identified during the health status assessment. Assessment is followed by the provision of medical advice or services, or referral to an appropriate specialist.
4. *Lifestyle counseling and health education* services are provided in individual and group settings by physiotherapist, dietitians, psychologists, and public health specialists to address risk factors, increase health literacy, as well as compliance and adherence to medical and health advice.
5. *Chronic care was reoriented focusing on rehabilitation* relying on other cluster workers, primarily physiotherapists. GPs refer their patients in need of rehabilitative services to local service providers, which is especially important for low-income patients many of whom cannot afford the costs of travel that need to be covered by the patients even in cases of free service.

Detailed evaluation of the effectiveness of these new services and their impact on the provision of PEs will be possible after the closure of the Model Programme in 2017.

## Discussion

Our study investigated the provision of preventive services in primary care as specified by a legal instrument in Hungary. The legal instrument in the form of a ministerial decree specifies the PEs, target groups, and their frequency hereby defining the reference intensity of preventive service use. Of these, 12 examinations were selected, and their non-provision was examined in both genders, three age groups, and four educational categories, based on a representative sample of the Hungarian adult population. As we have shown in our analysis, the provision of the selected PEs has been highly insufficient in the population and even more so in the population living in the most deprived regions of the country and in those targeted by the Primary Health Care Model Programme.

As opposed to our hypothesis, rates of non-performed preventive services are more frequent in females and in the oldest age group, that is, in those over 65 years of age. In alignment with our expectation, the rate of non-performed preventive services was highest among those with no more than primary education.

We postulated that preventive services are not performed due to their high cost to the provider, but this was not confirmed by analyzing the correlation between unit costs and the rates of service use either considering the total sample or various educational categories. In other words, economic constraints do not explain the high rates of non-provided examinations (29). Overall, approximately 31 million euros are saved per year by not paying for PEs. However, saved expenses are distributed in an unequal manner and are the largest among those in the lowest educational category.

As it is demonstrated by our analysis, neither the rates of not performed examinations per person per year nor their expenses are particularly high. It cannot be a significant determinant for the low rate of preventive services. What are missing are the structures that would be capable of providing these preventive services that have been legally specified for almost two decades and the financing system that motivates health-care (especially PHC) providers to deliver preventive services. Our results lead to the conclusion that a major point of consideration in redesigning the structure should be the adjustment of services taking into account the educational and age gradients of the population. Such developments have been tested in the outsourcing of post-socialist national health systems in the independent republics of the former Yugoslavia (30), sharing a similar historical legacy.

### Reorientation of Primary Care into Public Health Services

The principles of the GPs’ clusters’ organization and activities in the Model Programme was laid down in the Operations Manual finalized by May 2013 (31). The General Code of Practice was accepted in 2014; the Code of Practice for Asset Management was finalized in 2015. The website of the Programme was set up in 2014, and it not only provides information for lay audiences but is also used as a forum for information exchange for professionals involved in the Programme (32). The Operations Manual specified all PEs (including the 12 examined in this report) that are offered for all those adults who are registered with GPs working in the Model Programme.

The Primary Health Care Model Programme is established on the principles of the universal model of health care that was proposed in the seminal document, the Alma Ata Declaration (33) of the World Health Organization in 1978. This model defined PHC as an essential health care that should address the main health problems in the community by offering health promoting, disease preventing, curative, and rehabilitative services alike in the framework of a comprehensive national health system based on a wide societal allegiance. US authors proposed another selective model of health care that would target only diseases that affect many people by means for which proven and cost-effective treatment modalities are available (34). The latter model, originally proposed for developing countries, has become widespread in many developed countries as well (35). Rising public expectations for increasing well-being and helping people achieve good health led to the development of new public health and the health promotion movement (36) that aimed at providing interventions and services that selective PHC could not address.

The World Health Organization remained dedicated to the universal model of PHC attested by the World Health Report of 2000 that defined health systems as “all the activities whose primary purpose is to promote, restore or maintain health” (37). This report de-emphasized the traditional distinction between preventive (community-oriented, public health-type) and curative (individual-focused, clinical-type) activities and instead called for improved performance that delivers cost-effective interventions to the largest possible number of people. Measurement of health systems performance had been enabled by the Global Burden of Disease (GBD) project commissioned by the World Bank in 1990 that was later institutionalized at the WHO (38). The first GBD data generated by the Disease Burden Unit of the WHO were published in the aforementioned World Health Report.

The World Health Report 2008 reaffirmed the WHO’s stance on primary care, acknowledging the growing expectations for PHC in modern societies. In order to meet these expectations, the WHO called for reforms that *integrate public health actions with primary care* under the umbrella of intersectoral healthy public policies, coupled with reforms to deliver socially relevant and responsive service for everyone – guided by inclusive, participatory, and democratic stewardship (39). This conceptually remodeled stewardship requires an adjusted management accepting the same values and implementing those integrated services that are envisioned in line with the WHO call for reform. The GPs’ cluster Model Programme provides a working example not only for the integrated governance but also for the appropriately adjusted management of public health focused primary care.

### Financing Primary Care Services by Considering Performance Indicators

The financing of primary care is based worldwide on capitation, on performance-based reimbursement scheme, or on a combination of the two in a “mixed” form. Capitation-based financing (40) should not be regarded as essentially incorrect. An analysis carried out in the US to compare capitation- and performance-based funding schemes extended on providers and patients revealed no significant differences in characteristics of the care (41). Moreover, in many countries (e.g., the United Kingdom), capitation-based payment is under fine tuning rather than being rejected. The Hungarian practice has been burdened by several problems, some of which can be judged as barriers to fine tuning. A wide range of preventive services are defined in the legislative framework, but the list of services to be provided in primary care can be considered neither final nor based on consensus. In the present arrangement that trusts the client to request preventive services, the GP determines the range of preventive services he/she provides, not having to face any professional, legal, and financial consequences doing otherwise. However, performance results presented in our paper along with health economic considerations call for a move toward performance-based mixed financing (42). Restructuring primary care cannot be separated from the development of a system of performance indicators also accounting for financial consequences. The funding of preventive services in primary care is an international practice, proven by the service spectrum of the largest international health insurance companies (43). Medicare in the USA finances (44) adult vaccination (influenza, pneumococcus, and hepatitis B) programs, complex support of smoking cessation, and nutritional and dietary counseling in the frame of primary prevention, whereas screening for breast, cervical, and prostate cancer, cardiovascular diseases, diabetes, osteoporosis, and glaucoma are classified as secondary prevention; programs related to tertiary care are also funded. Funding of these services is not a charitable activity but a cost-reducing measure that helps avoid or postpone chronic diseases that place a severe burden on care. A mixed – capitation-based and fee-for service – primary care financing could make health promotion and prevention interventions more efficient. Therefore, we propose the introduction of a fee-for-service component for PHC in addition to the capitation-based financing, based on indicators to account for the preventive interventions performed by GPs (45).

## Conclusion

The Primary Health Care Model Programme of Hungary, by the support of the Swiss Government, constitutes the so-far largest-scale effort to redefine and expand the focus of the PHC system in Hungary. It has proved to be a heroic act because of its sheer scale and also because it had no precedence in the Hungarian health-care system. The Programme created a pressure for collaborative work, consultation, and engagement at each level from the GPs and health mediators up to the consortial level that has been clearly beneficial in terms of experience at the leadership, management, and field worker levels as well. The Programme has been able to mobilize the population for health services in very disadvantaged parts of the country. This has been shown by the fact that more than 80% of the population included in the Programme has already participated in health status assessment followed by medical risk assessment, lifestyle counseling, and/or chronic care as needed. These services include all 12 examinations covered in this report.

The Model Programme will come to an end in December 2016 so that a full evaluation will only be forthcoming afterward. However, it can already be stated that the Programme has produced a reformed model for service delivery and created a momentum that will hopefully turn into a policy decision to reorientate the entirety of PHC in Hungary in order to extend lives by providing services rather than saving expenses.

## Author Contributions

JS collected and prepared data as well as carried out the data analysis, contributing to their interpretation and drafting of the manuscript. KK participated in interpreting the data and drafting and finalizing the manuscript. MP and GF contributed to data collection and finalization of the manuscript. LK provided data from the National Health Insurance Fund and participated in data interpretation and drafting the manuscript. MJ participated in interpreting the data and drafting and finalizing the manuscript. RÁ drafted the concept of the paper as well as participated in interpreting results and drafting and finalizing the manuscript.

## Conflict of Interest Statement

The authors with the exception of MJ are contributors in various functions of the “Public Health Focused Model Programme for Organising Primary Care Services Backed by a Virtual Care Service Centre.” JS is in charge of the Research Workgroup of the Programme; KK is in charge of Roma targeted interventions of the Programme; MP is professional coordinator of the Programme; GF is assistant to the professional coordinator; LK is expert delegate of the National Health Insurance Fund; and RÁ is Chief Scientific Adviser of the Programme and in charge of the education compartment.
