# Supplementary material for: Capitation-Based Financing Hampers the Provision of Preventive Services in Primary Health Care
Source: Front Public Health. 2016 Sep 13;4:200. doi: 10.3389/fpubh.2016.00200 (PMC5020077; doi:10.3389/fpubh.2016.00200)
Supplement: Supplementary file 1 [file Table_1.DOCX]

Supplementary table: Samples and population by sex-age-education

|  |  |  | Primary or less | Vocational training | High school | Higher education | Total |
| --- | --- | --- | --- | --- | --- | --- | --- |
| intervention area | male | 20-39 | 50 (5,1%) | 82 (8,3%) | 29 (2,9%) | 13 (1,3%) | 174 (17,7%) |
|  |  | 40-64 | 40 (4,1%) | 121 (12,3%) | 16 (1,6%) | 17 (1,7%) | 194 (19,7%) |
|  |  | 65-X | 32 (3,2%) | 31 (3,1%) | 3 (0,3%) | 7 (0,7%) | 73 (7,4%) |
|  |  | **total** | **122 (12,4%)** | **234 (23,8%)** | **48 (4,9%)** | **37 (3,8%)** | **441 (44,8%)** |
|  | female | 20-39 | 61 (6,2%) | 70 (7,1%) | 37 (3,8%) | 24 (2,4%) | 192 (19,5%) |
|  |  | 40-64 | 81 (8,2%) | 80 (8,1%) | 36 (3,7%) | 27 (2,7%) | 224 (22,7%) |
|  |  | 65-X | 93 (9,4%) | 21 (2,1%) | 11 (1,1%) | 3 (0,3%) | 128 (13%) |
|  |  | **total** | **235 (23,9%)** | **171 (17,4%)** | **84 (8,5%)** | **54 (5,5%)** | **544 (55,2%)** |
|  | **both** |  | **357 (36,2%)** | **405 (41,1%)** | **132 (13,4%)** | **91 (9,2%)** | **985 (100%)** |
| study area | male | 20-39 | 140 (3,5%) | 341 (8,4%) | 113 (2,8%) | 84 (2,1%) | 678 (16,7%) |
|  |  | 40-64 | 185 (4,6%) | 526 (13%) | 92 (2,3%) | 89 (2,2%) | 892 (22%) |
|  |  | 65-X | 127 (3,1%) | 115 (2,8%) | 24 (0,6%) | 46 (1,1%) | 312 (7,7%) |
|  |  | **total** | **452 (11,2%)** | **982 (24,3%)** | **229 (5,7%)** | **219 (5,4%)** | **1882 (46,5%)** |
|  | female | 20-39 | 139 (3,4%) | 241 (6%) | 150 (3,7%) | 127 (3,1%) | 657 (16,2%) |
|  |  | 40-64 | 262 (6,5%) | 349 (8,6%) | 206 (5,1%) | 135 (3,3%) | 952 (23,5%) |
|  |  | 65-X | 347 (8,6%) | 101 (2,5%) | 75 (1,9%) | 34 (0,8%) | 557 (13,8%) |
|  |  | **total** | **748 (18,5%)** | **691 (17,1%)** | **431 (10,6%)** | **296 (7,3%)** | **2166 (53,5%)** |
|  | **both** |  | **1200 (29,6%)** | **1673 (41,3%)** | **660 (16,3%)** | **515 (12,7%)** | **4048 (100%)** |
| whole country | male | 20-39 | 227258 (2,9%) | 428042 (5,4%) | 505024 (6,4%) | 265141 (3,4%) | 1425465 (18,1%) |
|  |  | 40-64 | 280218 (3,5%) | 673474 (8,5%) | 408870 (5,2%) | 265576 (3,4%) | 1628138 (20,6%) |
|  |  | 65-X | 323245 (4,1%) | 55032 (0,7%) | 124328 (1,6%) | 114951 (1,5%) | 617556 (7,8%) |
|  |  | **total** | **830721 (10,5%)** | **1156548 (14,6%)** | **1038222 (13,1%)** | **645668 (8,2%)** | **3671159 (46,5%)** |
|  | female | 20-39 | 182168 (2,3%) | 244970 (3,1%) | 543770 (6,9%) | 414076 (5,2%) | 1384984 (17,5%) |
|  |  | 40-64 | 463751 (5,9%) | 359461 (4,6%) | 619805 (7,8%) | 337711 (4,3%) | 1780728 (22,6%) |
|  |  | 65-X | 737689 (9,3%) | 29021 (0,4%) | 202287 (2,6%) | 90567 (1,1%) | 1059564 (13,4%) |
|  |  | **total** | **1383608 (17,5%)** | **633452 (8%)** | **1365862 (17,3%)** | **842354 (10,7%)** | **4225276 (53,5%)** |
|  | **both** |  | **2214329 (28%)** | **1790000 (22,7%)** | **2404084 (30,4%)** | **1488022 (18,8%)** | **7896435 (100%)** |
